# American Academy of Dental Science

**Published:** 1891-05

**Authors:** 

**Affiliations:** American Academy Dental Science


### AMERICAN ACADEMY OF DENTAL SCIENCE.

The American Academy of Dental Science held its regular monthly meeting, January 7, 1891, in the Boston Medical Library Association rooms, President Seabury in the chair.

The paper for the evening was read by Jacob L. Williams, M.D. Subject: "Plastic Materials in the Preparatory Treatment of Teeth."

(For Dr. Williams's paper, see p. 281.)

*Dr. Chandler.*—I would like to ask the essayist what is the advantage of oxide of zinc, finely pulverized, over feldspar? Why not mix that in place of the oxide of zinc?

*Dr. Williams.*—I found that the oxides, especially the oxide of tin, made a more intimate combination with gutta-percha than any of the simple pulverized minerals like spar. We did at first mix a little spar, thinking to make a stiffer mass, and thereby counteract the stringiness of the gutta-percha. I found, however, that the spar made the filling more friable. It was very much like trying to make putty with chalk. It can be done in a way, but an oxide will make a harder material than any simple substance like white feldspar, or even clay. Oxide of tin was found to be too dark in color, and often discolored the teeth, though I think it had a more antiseptic effect than oxide of zinc. I took precautions, however, to insure the aseptic condition of cavities without regard to the material used for stopping. Oxide of zinc combined nicely with gutta-percha, at the same time making a white filling and one which would not discolor the teeth.

*Dr. Andrews.*—I think it is twelve years ago that I read a paper before the Massachusetts Dental Society on preparatory filling. I have the paper at home, and wish I had brought it with me that I might speak of some of the points contained in it. My idea was to prepare young teeth for future permanent fillings by using adhesive plastic stoppings. And I stated that I found some advantage in using lacto-phosphate of lime under such fillings in order to induce the pulp to throw off calcifying material, and thus make the teeth more dense. I was pretty heavily sat down upon by Dr. Chandler and other members present. Dr. Chandler did not then believe that there was an advantage in preparing frail young teeth so as to bear permanent fillings.

I believe that under adhesive fillings the tubuli are partially recalcified.

Dr. Black and others will tell us that the tubuli are filled with fat. I believe that the material which they call fat consists of minute globules, to which have been given the name of calcoglobulin, and these become calcified. I believe most heartily in preparatory fillings.

*Dr. Chandler.*—I remember the circumstance to which Dr. Andrews alludes. I did not then believe that lacto-phosphate of lime had any special virtue, and I do not believe it now. As I remember it, he made the statement that the lacto-phosphate of lime had some chemical or physiological action on the inside of the tooth which modified its condition and improved its quality. The ground that I took was that the simple filling of the tooth with gutta-percha stopped the fluids outside, arrested the ruin that was going on, and left the inside to take care of itself. It prevented all outside irritation and permitted the odontoblasts to go on with their natural work of depositing secondary dentine, but the filling had no chemical action, or any other influence with the tooth except the exclusion of destructive oral fluids.

*Dr. Andrews.*—I wish to correct the gentleman on one point. It was not gutta-percha which was used at that time, but oxychloride of zinc. The same results might, however, be obtained under gutta-percha if the same treatment were employed, provided the filling be absolutely tight. I will cite an instance showing the value of this preparatory filling. A student of mine had a pulp which was clearly bare, showing the pulsations of the vessels. An article which I had somewhere read stated that if lacto-phosphate of lime were placed over a pulp it would cause that organ to throw off calcifying material, thus forming a new covering. I had never as yet tried this method, but I now used it upon my student. The application was made, and an oxychloride filling inserted and kept there for about two years. At the end of that time, the filling, being worn down, was taken out, and a complete covering of dentine was found over the pulp. A gold filling was then inserted by Dr. I. J. Wetherbee, using the hand-mallet. The filling was made at a clinic before the Massachusetts Dental Society about ten years ago. I believe the filling is still in position, and the pulp still alive.

*Dr. Tucker.*—Some ten or twelve years ago I was filling a cuspid tooth for one of my own family. It was a large cavity and the pulp was nearly bare, so that you could distinctly see the pulsation. I filled it with gutta-percha, Hill's stopping, and allowed it to remain there for twelve months. Of course I had a chance to see it whenever I pleased, and I know that there was never any trouble or inflammation about it. At the end of the twelve months I removed the filling, and at the bottom of the cavity there was a hard polished substance which was protecting the pulp. I then filled the tooth with gold, and it has remained perfectly well and healthy these twelve years.

*President Seabury.*—Did the pulp bleed any?

*Dr. Tucker.*—Not at all. It was apparently in good condition, but you could plainly see the pulsation before using the gutta-percha.

*Dr. Williams.*—Some years ago, when lactophosphate of lime was suggested as a nutritive help to pulps in forming secondary dentine, I tried it for a while in several cases. The impression which I got—I will not say that I established it as a scientific fact—was that it favored the formation of nodules in the pulp rather than the formation of an even layer over the most exposed point. Several cases of exposed pulps which I treated with lactophosphate of lime I afterwards found had pulp-stones in them, and for this reason I left off using it.

*Dr. Briggs.*—A great deal of credit is due to Dr. Williams for his efforts to establish the efficacy of preparatory fillings in the treatment of teeth. Dr. Andrews speaks of the criticism to which the method was subjected twelve years ago. I was in the way of hearing those criticisms, and I am now very much pleased to congratulate Dr. Williams on having lived to see his theory placed on a footing where it cannot be criticised, and to see the profession cordially endorsing it.

*President Seabury.*—As the next thing on the programme, Dr. Andrews will show his improved saliva-ejector.

*Dr. Andrews.*—I suppose that most of us have had the same trouble with the ordinary saliva-ejector that I have had. I refer to the disagreeable sucking noise and to the pain that is caused to the patient by having the mucous membrane sucked into the slits or openings in the mouth-piece, sometimes causing a painful bruise. I have devised an appliance which has relieved me of all this trouble. It is very simple, and I cheerfully give it to the members of the profession. A small tube, open at the upper end, is soldered to the main tube, and opens into the bulb at the end of the mouth-piece a little lower than the openings in which the saliva is taken into the pipe. It is impossible for the mucous membrane to be sucked into these, as the air coming down this small tube would immediately release it. The one that I show you is rather a clumsy one, but works very perfectly in the mouth. Any brass-worker can make them.

*President Seabury.*—Cannot you alleviate that trouble by shutting off the water and making the suction less?

*Dr. Andrews.*—No; I have tried running the water slowly, but if there is suction enough to take the water in at all, it will also suck in the mucous membrane.

*President Seabury.*—I have an ejector designed by old Dr. Fisk when he first invented the saliva-ejector. By slowing the water the sucking noise will stop, and yet there is force enough to remove the saliva.

*Dr. Williams.*—I want to mention a shape designed by Dr. Merrill, an associate of mine. It is rounded like a ball, but turning in with a slight cup around the tube which comes up in the centre. The holes, instead of being in the side of the tube, are in the top of the cup, and the tissues are not liable to be drawn into them. I like its working very much.

*Dr. Eames.*—At the last meeting I read a paper on "Sensitive Dentine," advocating the use of warm air as an obtundent. The appliance I then had for warming the air being open to many objections, I have since experimented with small metal tubing, and as a result this little contrivance is now presented for your inspection. I have been using it successfully, both with the compressed air-tank of Codman & Shurtleff and with the common foot-bellows.

The tubing is coiled closely in this shape, and is connected with the foot-bellows or air-tank, and placed on the rim of the bracket-lamp. To the delivery-end is connected a nozzle of glass, containing a thermometer which indicates the temperature of the air very near the point of exit.

This is essential, as the air cools very rapidly in travelling a short distance.

My main point was the advocacy of warm air at a certain definite temperature; and by means of this instrument one can judge very nearly what the temperature of the air is when it strikes the tooth.

There is no need of freezing a tooth or boiling it, when 115° F. effectually obtunds the sensitiveness of dentine. During the past three or four weeks I have been using warm air exclusive of other means or medication, and it has been successful in every case thus far.

At the present time I allow the temperature, as indicated by the thermometer in the glass tube, to range from 115° to 125° F., using my best judgment as to the temperature of the tooth-structure itself. This is modified, of course, by the nearness with which the point is held to the tooth.

I aim to have the temperature such that I can hold the nozzle in contact with the tooth.

*Dr. Fillebrown.*—There is already in use a gas-burner which automatically controls the flame and regulates it to any desired temperature. This might be used under a small tank of water in which a coil of pipe is immersed. The air passing through the coil would be heated to any desired temperature.

*Dr. Eames.*—The air will cool before it reaches the mouth.

*Dr. Fillebrown.*—You can have the coil as near the mouth as you wish, and if necessary allow for the cooling of the air.

William H. Potter, D.M.D.,
*Editor American Academy Dental Science.*

